# Importance of ventilation and occupancy to *Mycobacterium tuberculosis* transmission rates in congregate settings

**DOI:** 10.1186/s12889-022-14133-5

**Published:** 2022-09-19

**Authors:** A. K. Deol, N. Shaikh, K. Middelkoop, M. Mohlamonyane, R. G. White, N. McCreesh

**Affiliations:** 1 Department of Infectious Disease Epidemiology, London School of Hygiene and Tropical Medicine, London, UK; 2 The Desmond Tutu HIV Centre, Institute of Infectious Disease and Molecular Medicine and Department of Medicine, University of Cape Town, Cape Town, South Africa

**Keywords:** Tuberculosis, Airborne transmission, Ventilation, Infection control, South Africa

## Abstract

**Background:**

Ventilation rates are a key determinant of the transmission rate of *Mycobacterium tuberculosis* and other airborne infections. Targeting infection prevention and control (IPC) interventions at locations where ventilation rates are low and occupancy high could be a highly effective intervention strategy. Despite this, few data are available on ventilation rates and occupancy in congregate locations in high tuberculosis burden settings.

**Methods:**

We collected carbon dioxide concentration and occupancy data in congregate locations and public transport on 88 occasions, in Cape Town, South Africa. For each location, we estimated ventilation rates and the relative rate of infection, accounting for ventilation rates and occupancy.

**Results:**

We show that the estimated potential transmission rate in congregate settings and public transport varies greatly between different settings. Overall, in the community we studied, estimated infection risk was higher in minibus taxis and trains than in salons, bars, and shops. Despite good levels of ventilation, infection risk could be high in the clinic due to high occupancy levels.

**Conclusion:**

Public transport in particular may be promising targets for infection prevention and control interventions in this setting, both to reduce *Mtb* transmission, but also to reduce the transmission of other airborne pathogens such as measles and SARS-CoV-2.

**Supplementary Information:**

The online version contains supplementary material available at 10.1186/s12889-022-14133-5.

## Introduction

Tuberculosis (TB) continues to pose a large public health burden, particularly in low- and middle-income settings with a high HIV prevalence. In 2020, TB claimed over 1.3 million lives worldwide [1]. While estimated global TB incidence fell by 9% between 2015 and 2019, the reductions were not large enough to meet the World Health Organization End TB Strategy goals of a 20% reduction by 2020, and 50% reduction by 2030 and by 2020 reductions had been slowed down to almost a complete stop due to the ongoing COVID-19 pandemic (and projected to worsen in the coming years) [1, 2].

Prevention and control measures currently focus mainly on biomedical interventions aimed at reducing the duration of infectiousness, such as case finding. A strong case has been made to also consider the potential reductions in TB incidence that could be achieved through reducing the rate of transmission of *Mycobacterium tuberculosis* (*Mtb)*, through improving ventilation rates in buildings and transport [3, 4]. The COVID-19 pandemic makes this call for increased attention to, and improvements in, ventilation particularly relevant, as it has become clear that airborne transmission also plays an important role in SARS-CoV-2 transmission [5].

Ventilation rates are a key determinant of rates of *Mtb* transmission, with a doubling in ventilation rates resulting in a halving of transmission rates [6]. Making simple, low-cost changes to building structures has been shown to be a highly effective way of improving ventilation rates, and therefore reducing transmission risk, in hospitals [7] and university buildings [8]. Even just opening windows and doors can dramatically increase ventilation, resulting in a nearly 7-fold increase in ventilation rates in houses in rural KwaZulu-Natal, South Africa [9]. Where improvements in ventilation rates are not possible, germicidal ultraviolet (GUV) disinfection systems have been shown to reduce the rate of *Mtb* transmission to guinea pigs by 70% [10].

Developing an understanding of the types of location where rates of transmission are highest is vital in maximising the impact of infection prevention and control interventions. *Mtb* is an airborne infection, and therefore transmission is believed to occur mainly indoors [3]. Data from molecular studies suggest that, in high incidence settings, only around 8–19% of tuberculosis results from transmission between household members [11], or between known close contacts [12]. This suggests that a large proportion of tuberculosis results from transmission in indoor congregate settings, such as clinics, schools, public transport, workplaces, or churches [4, 13, 14].

At present, very limited data are available on ventilation rates in congregate settings in areas with high incidences of TB. Taylor et al (2016) estimated ventilation rates in eight buildings in KwaZulu-Natal, South Africa, and concluded that *Mtb* transmission rates could be high in some buildings [15]. Andrews et al (2013) recorded ventilation rates on approximately 30 journeys by public transport in Cape Town, South Africa, concluding that daily commuters could have an annual risk of infection from public transport alone of 3.5–5% [14]. Finally, Andrews et al (2014) [16] and Patterson et al (2017) [17] recorded CO_2_ levels in congregate locations in Cape Town, concluding that schools are likely to be important sites of transmission for adolescents. CO_2_ levels provide a proxy for the level of transmission risk in a location, that can be useful for many applications, however occupancy data are also needed to estimate ventilation rates.

In this study, we start to fill this critical information gap, describing data collection and estimating ventilation rates, occupancy, and relative transmission rates on 37 building-days in total in congregate locations in the community; and 50 public transport vehicles, in Cape Town, South Africa.

## Methods

### Data collection

Our study site for data collection was a peri-urban township in Cape Town, South Africa.

This study site was chosen due to its high prevalence of TB and HIV [18]. Data collection was conducted over 15 months in 2018 and 2019 over which period we collected people count and CO_2_ data from 37 buildings (clinics, shops, churches, bars, and hair salons) and 50 different vehicle journeys across a range of public transport options: minibus taxis, buses, and trains (Table 1). A vehicle journey was defined as the period of movement of a passenger vehicle from point A to point B, with the start and end locations noted during data collection. Buildings and vehicles were chosen by convenience sampling, with the number of episodes of data collection in each vehicle and building type constrained by logistical issues including difficulties obtaining permission from building owners, public transport strikes, and safety issues.

**Table 1:** Building/vehicle type, number of buildings/vehicles, duration of collection, and estimated ventilation rates

Building / vehicle type	Number of data collection days (buildings) or journeys (transport)	Number of individual buildings	Total duration (building/vehicle-minutes)	Range of individual building/vehicle data collection period (minutes)	Estimated ventilation rates (litres per second / ls^−1^)
Church	6	5	191	15–55	116–5374
Clinic	4	1	142	23–58	671–1270
Salon	6	2	121	12–33	101–364
Bars	9	2	209	19–31	24–425
Shop	12	6	228	14–22	59–416
Bus	3	–	83	18–45	96–307
Train	4	–	373	64–103	232–2496
Taxi	43	–	876	11–39	44–443

**Fig. 1:**
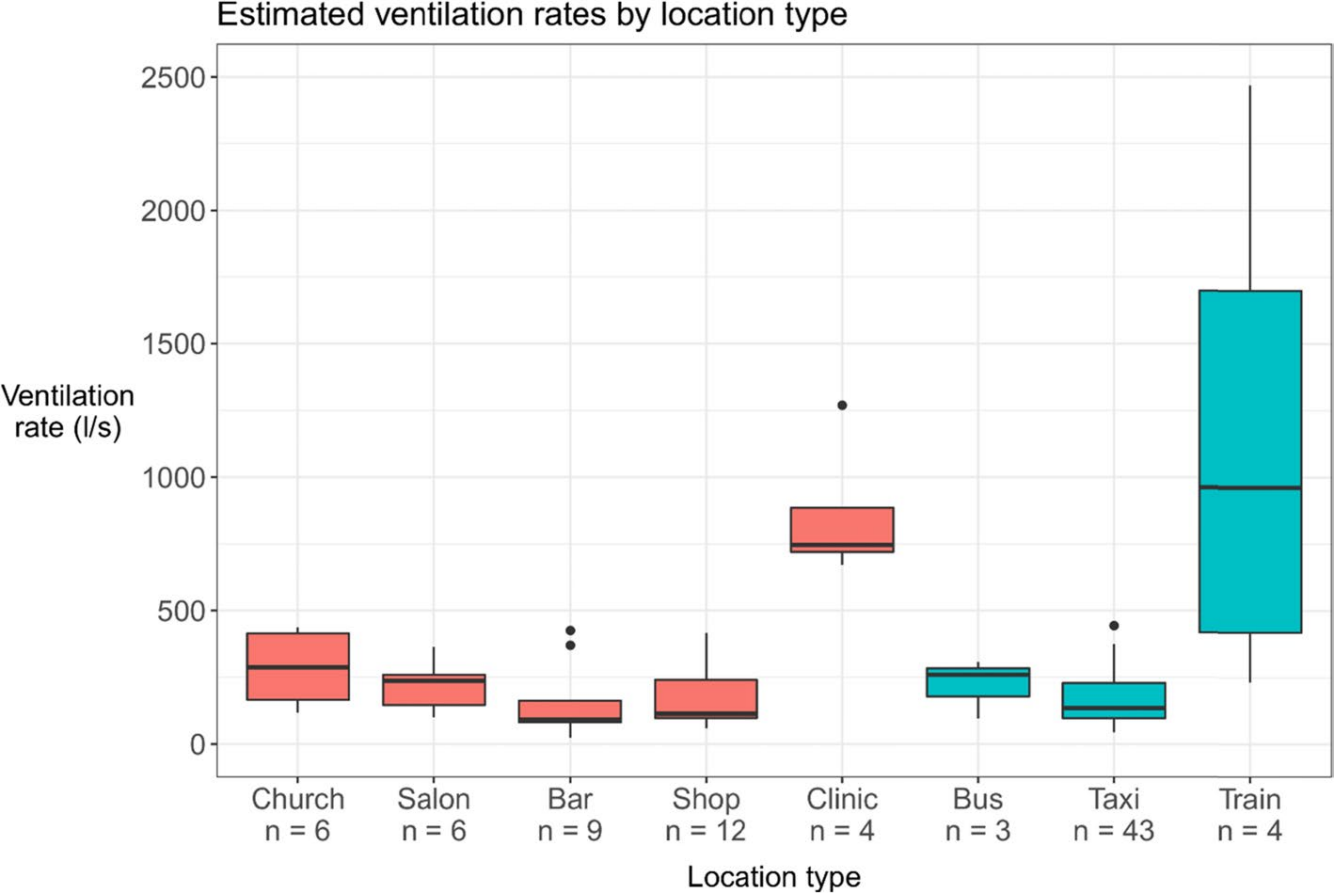
Estimated ventilation rates by building and vehicle type. The central line indicates the median, the box range the interquartile range (IQR), the whiskers the most extreme value within 1.5 * IQR from the box, and the points outlying values. The n’s indicate the number of collection days (buildings) or journeys (transport) One outlier point for a church (estimated ventilation rate of 5374 ls^− 1^) is not included in the figure

CO_2_ concentration was collected using CO_2_ dataloggers (CO2Meter, Inc., Model CM-0018, accuracy 30 ppm (±3% measured value)). Buildings were sampled for an average of 7.8 days each, excluding churches which were sampled for the duration of the church services (total of 191 minutes). The dataloggers were placed in the centre of the room inside the buildings and set to record the CO_2_ concentration every 5 minutes. The fieldworkers were positioned at the doorways of the buildings to collect people count data.

Public transport vehicles were sampled for the duration of the main routes run to and from the study community. Fieldworkers carried CO_2_ dataloggers on transport (outside of any bags, and away from the fieldworkers’ face) and the dataloggers were set to record CO_2_ concentration every 30 seconds. To collect people count data on trains, a fieldworker was positioned covering each pair of doors in the train carriage. Data were collected from a single carriage per train journey.

People count data were collected manually by fieldworkers using the Open Data Kit (ODK) software [19] on tablets. The ODK software allows for offline data collection with mobile devices in remote areas, with the ability to include GPS locations, and links to external datasets. Fieldworkers recorded the number of adults and children present in the location at the start of data collection and recorded each time an adult or child (estimated age < 15 years) entered or exited the building/vehicle. Times were recoded automatically by the software.

### Analysis

The ventilation rate was calculated using an approach based on that used by Persily and Jonge [20], modified to account for the non-steady states (in people counts and CO_2_ concentration). The total CO2 generation rate (G) was estimated by multiplying the number of individuals (n) in each age group at the same time point (t) by the corresponding G of the individuals, as referenced by Persily and Jonge [20]. We fitted a simple linear regression model for the relationship between the difference in CO_2_ concentration (C_in_ – C_out_) at each time point (Table 1) and the total CO_2_ generation rate at each time point (n*(t)*G), where the slope of the line determined the ventilation rate (Q). Further detailed equations for this methodology can be found in Deol et al (2021) [21].

The number of people in each location were age-differentiated (adult vs child), taking into account the age-related average CO_2_ generation rate (G) (assuming a MET of 2.0 which represents the activity of light slow walking on a level surface) [20].

‘Mean other people’ was calculated for each building/trip as a weighted average of the number of people present minus one, weighted according to the number of people present. In other words, it is a measure of the average other people present from the perspective of the people in the building/vehicle, similar to the approach used in estimating ‘shared air’ casual contacts in social contact studies [22]. We divided trips made on minibus taxis into ‘peak’ times, defined as 04:30–09:30 and then 16:30–18-30, and ‘off-peak’ at all other times.

To allow comparisons between different locations, we calculated a unitless ‘rate’ of potential transmission for each location, equal to the mean number of other people present (i.e., the mean number of people minus one) divided by the ventilation rate. The median potential transmission ‘rate’ across all locations was then calculated, and the rate relative to the median rate calculated for each location. This relative rate is proportional to risk estimated using the Wells-Riley equation [6], assuming the same prevalence of infectious tuberculosis in people in each building, and assuming that no saturation occurs.

## Results

Sixteen buildings were investigated for a total of 891 minutes and 134 transport journeys were measured. Data from 37 building-days and 50 journeys were used for this analysis. The remaining building days and transport journey data were omitted due to issues with dataloggers, including not recording start times or corrupted files due to faulty or damaged CO2 monitors (Table 1 and Supplementary Data table S1 and S2).

The estimated ventilation rates (in litres per second) for buildings ranged from 116 ls^− 1^ – 5374 ls^− 1^ for churches, 101 ls^− 1^ – 364 ls^− 1^ for hair salons, 24 ls^− 1^ – 425 ls^− 1^ for bars, 59 ls^− 1^ – 416 ls^− 1^ for shops, and 671 ls^− 1^ – 1270 ls^− 1^ for the clinic. For public transport, they ranged from 96 ls^− 1^ – 307 ls^− 1^ for buses, 44 ls^− 1^ – 443 ls^− 1^ for minibus taxis, and 232 ls^− 1^ – 2469 ls^− 1^ for trains (Fig. 1 and Supplementary Data tables S1 and S2).

**Fig. 2:**
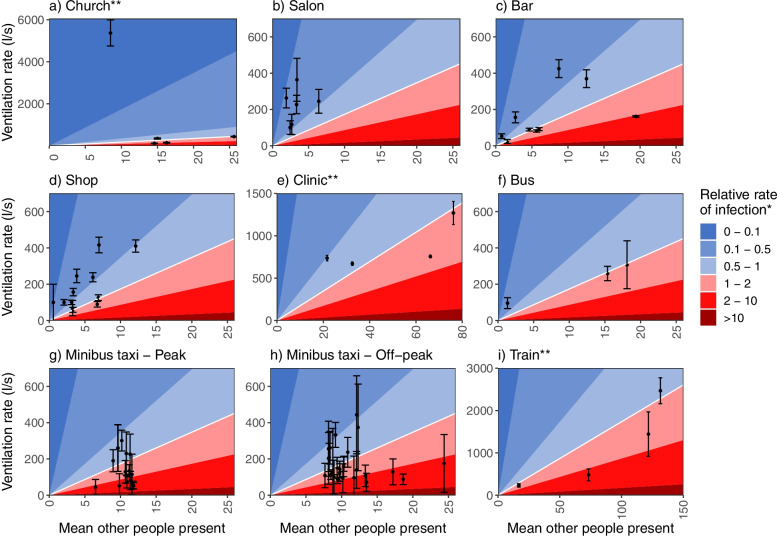
Estimated mean number of other people present, ventilation rate, and relative rate of potential transmission by building/transport type. Each point represents a single period of data collection in a building or vehicle, and shows the mean number of other people present, and the mean and 95% confidence intervals for the ventilation rates. For minibus taxis, the estimates are shown separately for peak times, defined as 04:30–09:30 and then 16:30–18-30, and off-peak at all other times. *To each person present, relative to the median rate of potential transmission across locations/transport, assuming the same prevalence of infectious tuberculosis in the other people present in the location/transport. **Note the different x-axis and y-axis scales

Figure 2 shows the estimated mean ventilation rates for each building observation or vehicle, plotted against the mean other people present. The contour lines give an indication of the estimated rate of potential transmission to each person present in the location, relative to the median estimated rate across all observations. They take into account ventilation rates and occupancy, and assume that the prevalence of people with infectious tuberculosis does not vary by location type.

Mean numbers of other people present were consistently low (≤25 people) in most location types. The exceptions was the clinic, where a mean of 76 other people were present during one period of data collection, and trains, where up to 132 mean other people were present. The estimated rate of potential transmission was highest on 12 of the 43 minibus taxis journeys, at up to 9 times the estimated median rate. Estimated rates were also consistently high in trains, with values close to or above the median on all four occasions. Estimated rates tended to be low, relative to the median, in salons, bars, and shops.

## Discussion

Our findings show that the risk of transmission per minute spent in congregate settings or on public transport varies greatly between different locations/transport types. High levels of variation were found both between different types of location and transport, and also between different buildings/transport of the same type. Nevertheless, our findings provide a clear indication, that in the community we studied, transmission risk is likely to be higher in minibus taxis and trains than in salons, bars, and shops. Public transport may therefore be promising targets for infection prevention and control interventions in this setting, both to reduce *Mtb* transmission, but also to reduce the transmission of other airborne pathogens such as measles and SAR-CoV-2.

The drivers of high relative rate of transmission varied between the two high risk transport types and suggest that different intervention strategies may be optimal in different types of location. In minibus taxis, transmission risk was predominantly driven by low ventilation levels. Guidelines and rules requiring some windows to remain open could greatly reduce risk, although they may be difficult to enforce. In trains, ventilation levels were more much more variable, ranging from 232 l^− 1^ – 2469 l^− 1^, with the number of people present being the main driver of transmission risk. Ensuring that windows are kept open may reduce risk, but even with adequate levels of ventilation, the very high numbers of people present in train carriages on some trips mean that risk is likely to remain high. Measures to reduce overcrowding on trains may be necessary to reduce risk to acceptable levels.

We estimated that ventilation rates were low in small, congregate buildings in the community such as hair salons and shops. As only small number of people were present at a time, however, the relative rate of potential transmission was low, and we do not consider these to be particularly high-risk locations. In contrast, while the ventilation rates in the (naturally ventilated) clinic were higher than in most other buildings (reflecting the larger size of the waiting area), estimated rates of potential transmission in the clinic were nevertheless high at times, due to high occupancy levels. While we only collected data from the single clinic that serves the study community, this finding can be cautiously generalised to many other clinics in South Africa, as overcrowding in public clinics is common [23]. An increased prevalence of people with undiagnosed tuberculosis in clinics may further increase risk [24, 25]. Unless very high ventilation rates can be achieved in clinics, or crowding reduced, other measures such as GUV systems may be desirable to reduce risk.

WHO IPC guidelines recommend that ventilation systems are used to reduce *Mtb* transmission in settings with a high risk of transmission, but do not give an explicit recommendation for a minimum acceptable ventilation rate. Our findings support this, as they demonstrate that risk varies greatly with the number of people present in a room, in addition to ventilation rates. Far higher ventilation rates will be needed to reduce transmission risk to acceptable levels in crowded locations than in quiet ones.

We presented our main results as estimated rate of transmission relative to the median rate across the locations we studied. These estimated ‘rates’ are correlated with the observed CO_2_ concentrations (see supplemental data), which can be used directly as a proxy for transmission risk if estimates of ventilation rates are not required. We chose not to use the Wells-Riley equation [6] to estimate absolute risk. This is because the Wells-Riley approach relies on an estimate of the rate of quanta production, about which there is considerable uncertainty, with estimates for tuberculosis ranging from 0.62–8.2 or more [26, 27] – a greater than 10-fold difference. Estimates of risk by building type calculated using the Wells-Riley approach are given in the supporting material.

We recorded data on more than 1 day in more than one salon, bar, and shop. While the numbers of repeat visits were too small for a formal statistical comparison to be adequately powered, the data show that the variation in the ventilation rates between the same building on different days was as large as the variation between different buildings of the same type (Table S1). This finding is not unexpected. Factors such as changes in outdoor wind speed or windows being opened or closed can have large effects on ventilation rates [9]. It presents challenges for data collection however, with recordings over a much larger number of days being needed to gain a full understanding of variation in ventilation rates. Ventilation rates may also have changed during data collection, particularly on transport where the speed changed and doors were opened and closed. Our estimated ventilation rates should be interpreted as average rates over the data collection period.

There are a number of limitations to our work. In estimating the relative rate of potential transmission in congregate locations, we assume that the prevalence of people with infectious tuberculosis is the same in all locations. This means that we may have underestimated the risk in the clinic, with the prevalence of tuberculosis in people attending clinics likely to be higher than the prevalence in many other locations [24]. We also do not take into account the age distribution of people present, and the fact that the prevalence of infectious tuberculosis is low in children [28]. We may therefore have underestimated the relative risk in bars, where the majority of people present were adults. The effect of varying prevalences of tuberculosis on estimated risk in other location types is unclear, however could be explored using mathematical modelling of social contact data. We may also have underestimated the relative rate of transmission in bars due to the timing of data collection, as safety concerns meant that we were unable to collect data at peak times on Friday and Saturday nights, when occupancy levels were likely to have been higher. Locations were selected by convenience sampling and therefore may not be fully representative of all locations, however they were chosen to cover a diverse range of locations (e.g. based on location size and structure). We sampled the only clinic in the community and approached owners or pastors of the other locations for permission to sample. Sampling was challenging: while most pastors and owners were happy for us to sample, and so a wide range of venues were sampled, counters often didn’t record or tampered with (batteries removed for example), and so the final sample represents those venues with viable data. Seasonality is likely to have an effect on ventilation rates, with rates likely to be lower in winter (and in summer in air conditioned buildings) when windows are more likely to be closed. The majority of our data collection in buildings and transport was conducted in summer and winter respectively (see supplementary data), and we may therefore have overestimated risk in transport relative to buildings. Finally, data on building/transport occupancy and ventilation levels should be combined with social contact or time use data to gain a more complete understanding of the likely contributions of different types of location/transport to overall *Mtb* transmission in the community.

We assumed that all people present in all settings had an activity level consistent with light slow walking on a level surface. If average activity levels were lower in a setting, for instance people sitting on a bus, then CO_2_ generation rates would have been lower, and we will have overestimated the ventilation rates. The reverse is also true, if activity levels were higher in a setting. Our estimates of relative risk by setting are robust to differences in CO_2_ generation levels however, as any under- or over-estimation of ventilation rates will be cancelled out by a corresponding over- or under-estimation of the risk associated with the number of other people present.

To conclude, we contribute knowledge to an important but neglected area of tuberculosis research, presenting estimates of ventilation rates and relative rates of potential transmission from a wide range of congregate settings in a high TB community in Cape Town, South Africa. We show that, in our setting, the rate of transmission is likely to be particularly high on public transport, making it a promising target for infection prevention and control interventions. Risk may also be high in clinics, even with high ventilation rates, due to high occupancy levels and an increased prevalence of people with infectious TB.

## Supplementary Information

**Additional file 1: Table S1**. Detailed results for each building. **Table S2**. Detailed results for each vehicle. Rebreathed air calculation. **Fig. S1**. Estimated transmission rate against mean CO2 concentrations by building and vehicle.

## Data Availability

The datasets supporting the conclusions of this article are available in the London School of Hygiene and Tropical Medicine Data Compass repository, 10.17037/DATA.00002427.
