# Supplementary material for: Importance of ventilation and occupancy to Mycobacterium tuberculosis transmission rates in congregate settings
Source: BMC Public Health. 2022 Sep 19;22:1772. doi: 10.1186/s12889-022-14133-5 (PMC9483862; doi:10.1186/s12889-022-14133-5)
Supplement: Supplementary file 1 — Additional file 1: Table S1. Detailed results for each building. Table S2. Detailed results for each vehicle. Rebreathed air calculation. Fig. S1. Estimated transmission rate against mean CO2 concentrations by building and vehicle. [file 12889_2022_14133_MOESM1_ESM.docx]

# Supplementary Data

**Table S1. Detailed results for each building**

| **Building type** | **Building** | **Date of collection** | **Day of week** | **Duration of collection (minutes)** | **Mean other people present** | **Rebreathed percentage (%)** | **Ventilation rate (95% CI) l/s** | **P from Wells-Riley (quanta=0.62)** | **P from Wells-Riley (quanta=8.2)** |
| --- | --- | --- | --- | --- | --- | --- | --- | --- | --- |
| Church | ChurchA | 11/08/2019 | Sunday | 36 | 15 | 1.1 | 116 (105 - 127) | 1.07E-04 (1.18E-04 - 9.75E-05) | 1.41E-03 (1.56E-03 - 1.29E-03) |
|  | ChurchB | 20/10/2019 | Sunday | 15 | 16 | 0.8 | 144 (122 - 165) | 9.19E-05 (1.08E-04 - 8.00E-05) | 1.22E-03 (1.43E-03 - 1.06E-03) |
|  | ChurchC | 20/10/2019 | Sunday | 22 | 26 | 0.5 | 437 (423 - 450) | 4.92E-05 (5.08E-05 - 4.77E-05) | 6.51E-04 (6.72E-04 - 6.31E-04) |
|  | ChurchD1 | 01/04/2019 | Monday | 31 | 0 | 0.0 | 228 (0 - 492) | 0 | 0 |
|  | ChurchD2 | 27/03/2019 | Wednesday | 32 | 8.5 | 0.0 | 5374 (4751 - 5997) | 1.31E-06 (1.48E-06 - 1.17E-06) | 1.73E-05 (1.96E-05 - 1.55E-05) |
|  | ChurchE | 28/07/2019 | Sunday | 55 | 15 | 0.4 | 346 (321 - 371) | 3.58E-05 (3.86E-05 - 3.34E-05) | 4.74E-04 (5.11E-04 - 4.42E-04) |
| Clinic | ClinicA1 | 10/07/2019 | Wednesday | 58 | 33 | 0.5 | 671 (650 - 691) | 4.07E-05 (4.19E-05 - 3.95E-05) | 5.38E-04 (5.55E-04 - 5.22E-04) |
|  | ClinicA2 | 11/07/2019 | Thursday | 23 | 76 | 0.5 | 1270 (1134 - 1405) | 4.95E-05 (5.54E-05 - 4.47E-05) | 6.54E-04 (7.32E-04 - 5.91E-04) |
|  | ClinicA3 | 12/07/2019 | Friday | 30 | 22 | 0.2 | 737 (705 - 769) | 2.47E-05 (2.58E-05 - 2.36E-05) | 3.26E-04 (3.41E-04 - 3.13E-04) |
|  | ClinicA4 | 15/07/2019 | Monday | 31 | 66 | 0.8 | 756 (741 - 772) | 7.21E-05 (7.37E-05 - 7.07E-05) | 9.54E-04 (9.74E-04 - 9.34E-04) |
| Salon | SalonA1 | 17/07/2019 | Wednesday | 26 | 3.4 | 0.1 | 364 (245 - 482) | 7.73E-06 (1.15E-05 - 5.83E-06) | 1.02E-04 (1.52E-04 - 7.71E-05) |
|  | SalonA2 | 18/07/2019 | Thursday | 12 | 1.9 | 0.1 | 263 (209 - 318) | 5.96E-06 (7.51E-06 - 4.95E-06) | 7.89E-05 (9.94E-05 - 6.54E-05) |
|  | SalonA3 | 19/07/2019 | Friday | 33 | 3.3 | 0.1 | 228 (177 - 278) | 1.20E-05 (1.54E-05 - 9.81E-06) | 1.59E-04 (2.04E-04 - 1.30E-04) |
|  | SalonB1 | 16/08/2019 | Friday | 19 | 6.4 | 0.2 | 245 (180 - 311) | 2.16E-05 (2.95E-05 - 1.70E-05) | 2.85E-04 (3.90E-04 - 2.25E-04) |
|  | SalonB2 | 20/08/2019 | Tuesday | 15 | 2.7 | 0.2 | 118 (62 - 173) | 1.90E-05 (3.61E-05 - 1.29E-05) | 2.51E-04 (4.78E-04 - 1.70E-04) |
|  | SalonB3 | 21/08/2019 | Wednesday | 16 | 2.4 | 0.2 | 101 (64 - 138) | 1.97E-05 (3.12E-05 - 1.44E-05) | 2.61E-04 (4.13E-04 - 1.91E-04) |
| Bar | BarA1 | 15/08/2019 | Thursday | 19 | 0.81 | 0.3 | 53 (40 - 66) | 1.26E-05 (1.67E-05 - 1.01E-05) | 1.67E-04 (2.20E-04 - 1.34E-04) |
|  | BarA2 | 15/08/2019 | Thursday | 31 | 8.7 | 0.2 | 425 (376 - 474) | 1.69E-05 (1.91E-05 - 1.52E-05) | 2.24E-04 (2.53E-04 - 2.01E-04) |
|  | BarA3 | 18/08/2019 | Sunday | 30 | 13 | 0.3 | 370 (321 - 419) | 2.91E-05 (3.35E-05 - 2.57E-05) | 3.84E-04 (4.43E-04 - 3.40E-04) |
|  | BarA4 | 20/08/2019 | Tuesday | 19 | 2.7 | 0.2 | 157 (127 - 187) | 1.42E-05 (1.76E-05 - 1.20E-05) | 1.88E-04 (2.33E-04 - 1.58E-04) |
|  | BarB1 | 03/07/2019 | Wednesday | 22 | 1.6 | 0.6 | 24 (13 - 34) | 5.60E-05 (9.91E-05 - 3.90E-05) | 7.40E-04 (1.31E-03 - 5.16E-04) |
|  | BarB2 | 04/07/2019 | Thursday | 22 | 6 | 0.7 | 92 (84 - 100) | 5.38E-05 (5.92E-05 - 4.94E-05) | 7.12E-04 (7.82E-04 - 6.53E-04) |
|  | BarB3 | 05/07/2019 | Friday | 20 | 5.7 | 0.7 | 82 (75 - 88) | 5.76E-05 (6.26E-05 - 5.34E-05) | 7.62E-04 (8.28E-04 - 7.06E-04) |
|  | BarB4 | 07/07/2019 | Sunday | 20 | 19 | 1.2 | 162 (157 - 166) | 9.71E-05 (9.98E-05 - 9.46E-05) | 1.28E-03 (1.32E-03 - 1.25E-03) |
|  | BarB5 | 09/07/2019 | Tuesday | 26 | 4.6 | 0.6 | 89 (82 - 97) | 4.25E-05 (4.65E-05 - 3.92E-05) | 5.62E-04 (6.15E-04 - 5.18E-04) |
| Shop | ShopA1 | 29/07/2019 | Monday | 20 | 3.9 | 0.1 | 245 (208 - 283) | 1.31E-05 (1.55E-05 - 1.14E-05) | 1.74E-04 (2.05E-04 - 1.51E-04) |
|  | ShopA2 | 30/07/2019 | Tuesday | 19 | 7 | 0.2 | 416 (373 - 459) | 1.39E-05 (1.55E-05 - 1.26E-05) | 1.84E-04 (2.05E-04 - 1.67E-04) |
|  | ShopA3 | 31/07/2019 | Wednesday | 20 | 3.1 | 0.4 | 100 (88 - 112) | 2.56E-05 (2.91E-05 - 2.29E-05) | 3.39E-04 (3.85E-04 - 3.02E-04) |
|  | ShopB1 | 19/09/2019 | Thursday | 22 | 6.7 | 0.5 | 89 (74 - 105) | 6.19E-05 (7.48E-05 - 5.28E-05) | 8.19E-04 (9.89E-04 - 6.99E-04) |
|  | ShopB2 | 20/09/2019 | Friday | 19 | 12 | 0.3 | 410 (377 - 444) | 2.42E-05 (2.63E-05 - 2.23E-05) | 3.20E-04 (3.48E-04 - 2.95E-04) |
|  | ShopC1 | 12/08/2019 | Monday | 19 | 3.3 | 0.3 | 69 (26 - 111) | 3.97E-05 (1.03E-04 - 2.46E-05) | 5.25E-04 (1.36E-03 - 3.25E-04) |
|  | ShopC2 | 13/08/2019 | Tuesday | 14 | 0.57 | 0.1 | 100 (0 - 200) | 4.71E-06 (0 - 2.35E-06) | 6.23E-05 (0- 3.11E-05) |
|  | ShopD1 | 04/09/2019 | Wednesday | 18 | 3.3 | 0.5 | 59 (45 - 72) | 4.63E-05 (6.01E-05 - 3.77E-05) | 6.13E-04 (7.95E-04 - 4.99E-04) |
|  | ShopD2 | 05/09/2019 | Thursday | 19 | 6.9 | 0.5 | 126 (110 - 142) | 4.54E-05 (5.21E-05 - 4.02E-05) | 6.00E-04 (6.88E-04 - 5.32E-04) |
|  | ShopE1 | 04/09/2019 | Wednesday | 21 | 6.1 | 0.2 | 238 (213 - 263) | 2.12E-05 (2.37E-05 - 1.92E-05) | 2.80E-04 (3.13E-04 - 2.53E-04) |
|  | ShopE2 | 05/09/2019 | Thursday | 20 | 3.4 | 0.2 | 157 (136 - 177) | 1.79E-05 (2.06E-05 - 1.59E-05) | 2.37E-04 (2.72E-04 - 2.10E-04) |
|  | ShopF1 | 18/10/2019 | Friday | 17 | 2.1 | 0.3 | 100 (85 - 115) | 1.73E-05 (2.05E-05 - 1.50E-05) | 2.29E-04 (2.71E-04 - 1.99E-04) |

**Table S2. Detailed results for each vehicle**

| **Type** | **Vehicle** | **Date** | **Day of week** | **Duration of collection (minutes)** | **Time** | **Mean other people present** | **Rebreathed percentage (%)** | **Ventilation rate (95% CI) l/s** | **P from Wells-Riley (quanta=0.62)** | **P from Wells-Riley (quanta=8.2)** |
| --- | --- | --- | --- | --- | --- | --- | --- | --- | --- | --- |
| Bus | Bus1a | 30/04/2019 | Saturday | 20 | Off-peak | 18 | 0.7 | 307 (175 - 439) | 4.84E-05 (8.49E-05 - 3.39E-05) | 6.41E-04 (1.12E-03 - 4.48E-04) |
|  | Bus1b | 30/04/2019 | Saturday | 45 | Off-peak | 15 | 1.2 | 259 (219 - 299) | 4.79E-05 (5.65E-05 - 4.15E-05) | 6.33E-04 (7.48E-04 - 5.49E-04) |
|  | Bus2 | 30/04/2019 | Saturday | 18 | Off-peak | 1.3 | 0.5 | 96 (66 - 126) | 1.12E-05 (1.63E-05 - 8.53E-06) | 1.48E-04 (2.16E-04 - 1.13E-04) |
| Minibus taxi | Taxi1 | 13/12/2018 | Friday | 22 | Off-peak | 7.7 | 1.2 | 109 (42 - 176) | 5.84E-05 (1.53E-04 - 3.61E-05) | 7.73E-04 (2.03E-03 - 4.77E-04) |
|  | Taxi10 | 29/11/2018 | Tuesday | 23 | Off-peak | 8.2 | 0.6 | 256 (105 - 408) | 2.65E-05 (6.49E-05 - 1.66E-05) | 3.50E-04 (8.57E-04 - 2.20E-04) |
|  | Taxi11a | 27/04/2019 | Tuesday | 17 | Peak | 11 | 2.4 | 73 (40 - 107) | 1.24E-04 (2.27E-04 - 8.52E-05) | 1.64E-03 (3.00E-03 - 1.13E-03) |
|  | Taxi11b | 27/04/2019 | Tuesday | 24 | Off-peak | 12 | 0.4 | 443 (229 - 658) | 2.24E-05 (4.34E-05 - 1.51E-05) | 2.96E-04 (5.74E-04 - 1.99E-04) |
|  | Taxi11c | 27/04/2019 | Tuesday | 21 | Off-peak | 8.5 | 3.0 | 114 (87 - 140) | 6.18E-05 (8.09E-05 - 5.00E-05) | 8.17E-04 (1.07E-03 - 6.61E-04) |
|  | Taxi12 | 13/12/2018 | Friday | 37 | Off-peak | 9.1 | 0.6 | 332 (264 - 401) | 2.26E-05 (2.85E-05 - 1.87E-05) | 2.99E-04 (3.78E-04 - 2.48E-04) |
|  | Taxi13 | 29/11/2018 | Tuesday | 37 | Off-peak | 10 | 1.2 | 174 (132 - 215) | 4.76E-05 (6.27E-05 - 3.84E-05) | 6.30E-04 (8.29E-04 - 5.08E-04) |
|  | Taxi14 | 13/12/2018 | Friday | 27 | Off-peak | 24 | 0.8 | 175 (15 - 335) | 1.13E-04 (1.29E-03 - 5.93E-05) | 1.50E-03 (1.69E-02 - 7.84E-04) |
|  | Taxi15 | 14/12/2018 | Saturday | 20 | Off-peak | 9.7 | 1.3 | 144 (101 - 188) | 5.56E-05 (7.96E-05 - 4.27E-05) | 7.35E-04 (1.05E-03 - 5.65E-04) |
|  | Taxi16a | 17/01/2019 | Friday | 15 | Peak | 11 | 2.1 | 81 (31 - 131) | 1.12E-04 (2.91E-04 - 6.93E-05) | 1.48E-03 (3.84E-03 - 9.16E-04) |
|  | Taxi16b | 17/01/2019 | Friday | 18 | Off-peak | 10 | 1.2 | 93 (14 - 173) | 8.86E-05 (6.01E-04 - 4.78E-05) | 1.17E-03 (7.93E-03 - 6.32E-04) |
|  | Taxi17 | 19/12/2018 | Friday | 12 | Off-peak | 17 | 1.8 | 129 (57 - 200) | 1.09E-04 (2.45E-04 - 7.02E-05) | 1.44E-03 (3.24E-03 - 9.28E-04) |
|  | Taxi18 | 23/01/2019 | Friday | 11 | Peak | 12 | 1.2 | 115 (3 - 226) | 8.65E-05 (3.28E-03 - 4.39E-05) | 1.14E-03 (4.25E-02 - 5.80E-04) |
|  | Taxi19a | 23/11/2018 | Monday | 26 | Off-peak | 12 | 1.1 | 96 (0 - 216) | 1.04E-04 (0- 4.60E-05) | 1.37E-03 (0- 6.08E-04) |
|  | Taxi19b | 23/11/2018 | Monday | 21 | Off-peak | 8.2 | 1.0 | 203 (60 - 346) | 3.34E-05 (1.12E-04 - 1.96E-05) | 4.41E-04 (1.48E-03 - 2.59E-04) |
|  | Taxi2 | 14/12/2018 | Saturday | 19 | Off-peak | 9.7 | 1.3 | 125 (77 - 173) | 6.41E-05 (1.04E-04 - 4.63E-05) | 8.48E-04 (1.38E-03 - 6.12E-04) |
|  | Taxi20 | 24/01/2019 | Saturday | 14 | Peak | 9.6 | 0.8 | 260 (131 - 389) | 3.05E-05 (6.05E-05 - 2.04E-05) | 4.04E-04 (7.99E-04 - 2.70E-04) |
|  | Taxi21 | 28/04/2019 | Thursday | 14 | Off-peak | 8.9 | 1.3 | 100 (7 - 193) | 7.37E-05 (1.10E-03 - 3.81E-05) | 9.74E-04 (1.45E-02 - 5.04E-04) |
|  | Taxi22 | 29/11/2018 | Tuesday | 17 | Off-peak | 13 | 2.4 | 71 (22 - 121) | 1.51E-04 (4.98E-04 - 8.87E-05) | 1.99E-03 (6.57E-03 - 1.17E-03) |
|  | Taxi23a | 27/04/2019 | Tuesday | 14 | Off-peak | 19 | 3.4 | 88 (59 - 116) | 1.79E-04 (2.67E-04 - 1.35E-04) | 2.37E-03 (3.52E-03 - 1.78E-03) |
|  | Taxi23b | 27/04/2019 | Tuesday | 17 | Peak | 11 | 0.9 | 227 (116 - 337) | 4.01E-05 (7.82E-05 - 2.70E-05) | 5.31E-04 (1.03E-03 - 3.57E-04) |
|  | Taxi23c | 27/04/2019 | Tuesday | 16 | Off-peak | 13 | 2.2 | 107 (48 - 166) | 1.01E-04 (2.26E-04 - 6.47E-05) | 1.33E-03 (2.98E-03 - 8.55E-04) |
|  | Taxi24a | 23/01/2019 | Friday | 23 | Peak | 11 | 1.1 | 230 (111 - 349) | 3.95E-05 (8.16E-05 - 2.61E-05) | 5.23E-04 (1.08E-03 - 3.45E-04) |
|  | Taxi24b | 23/01/2019 | Friday | 16 | Off-peak | 8.8 | 0.9 | 128 (0 - 369) | 5.68E-05 (0- 1.97E-05) | 7.51E-04 (0- 2.61E-04) |
|  | Taxi25 | 24/01/2019 | Saturday | 14 | Peak | 9.6 | 0.8 | 260 (131 - 389) | 3.05E-05 (6.05E-05 - 2.04E-05) | 4.04E-04 (7.99E-04 - 2.70E-04) |
|  | Taxi26 | 20/12/2018 | Sunday | 21 | Off-peak | 12 | 1.1 | 139 (38 - 241) | 7.12E-05 (2.64E-04 - 4.12E-05) | 9.42E-04 (3.49E-03 - 5.44E-04) |
|  | Taxi27 | 28/04/2019 | Thursday | 15 | Peak | 12 | 4.9 | 52 (34 - 70) | 1.91E-04 (2.92E-04 - 1.42E-04) | 2.52E-03 (3.85E-03 - 1.88E-03) |
|  | Taxi28 | 29/01/2019 | Saturday | 17 | Peak | 9.8 | 1.9 | 52 (0 - 118) | 1.55E-04 (0- 6.84E-05) | 2.05E-03 (0- 9.04E-04) |
|  | Taxi29b | 27/04/2019 | Tuesday | 19 | Off-peak | 12 | 0.5 | 374 (135 - 613) | 2.65E-05 (7.34E-05 - 1.62E-05) | 3.51E-04 (9.70E-04 - 2.14E-04) |
|  | Taxi29c | 27/04/2019 | Tuesday | 18 | Peak | 12 | 3.7 | 57 (36 - 78) | 1.75E-04 (2.79E-04 - 1.28E-04) | 2.31E-03 (3.69E-03 - 1.69E-03) |
|  | Taxi30 | 28/04/2019 | Thursday | 12 | Peak | 6.5 | 1.8 | 44 (2 - 87) | 1.21E-04 (2.68E-03 - 6.20E-05) | 1.60E-03 (3.49E-02 - 8.20E-04) |
|  | Taxi31 | 29/01/2019 | Saturday | 20 | Peak | 10 | 0.5 | 301 (244 - 358) | 2.75E-05 (3.39E-05 - 2.31E-05) | 3.63E-04 (4.48E-04 - 3.05E-04) |
|  | Taxi32 | 13/12/2018 | Friday | 39 | Off-peak | 9.4 | 1.3 | 152 (94 - 209) | 5.12E-05 (8.26E-05 - 3.71E-05) | 6.77E-04 (1.09E-03 - 4.91E-04) |
|  | Taxi33 | 29/11/2018 | Tuesday | 39 | Off-peak | 11 | 0.8 | 237 (155 - 319) | 3.84E-05 (5.88E-05 - 2.85E-05) | 5.08E-04 (7.77E-04 - 3.77E-04) |
|  | Taxi3a | 17/01/2019 | Friday | 24 | Peak | 11 | 1.8 | 129 (66 - 192) | 7.05E-05 (1.37E-04 - 4.74E-05) | 9.32E-04 (1.81E-03 - 6.27E-04) |
|  | Taxi3b | 17/01/2019 | Friday | 22 | Off-peak | 10 | 1.5 | 156 (102 - 211) | 5.28E-05 (8.10E-05 - 3.92E-05) | 6.99E-04 (1.07E-03 - 5.18E-04) |
|  | Taxi4 | 20/12/2018 | Sunday | 21 | Off-peak | 8.3 | 1.0 | 208 (131 - 286) | 3.29E-05 (5.25E-05 - 2.40E-05) | 4.35E-04 (6.94E-04 - 3.17E-04) |
|  | Taxi5 | 23/01/2019 | Friday | 20 | Peak | 11 | 1.9 | 134 (100 - 168) | 6.79E-05 (9.11E-05 - 5.41E-05) | 8.98E-04 (1.20E-03 - 7.16E-04) |
|  | Taxi6 | 23/11/2018 | Monday | 20 | Off-peak | 8.4 | 0.7 | 262 (171 - 352) | 2.65E-05 (4.05E-05 - 1.97E-05) | 3.51E-04 (5.35E-04 - 2.61E-04) |
|  | Taxi7 | 24/01/2019 | Saturday | 14 | Peak | 9.6 | 0.8 | 260 (131 - 389) | 3.05E-05 (6.05E-05 - 2.04E-05) | 4.04E-04 (7.99E-04 - 2.70E-04) |
|  | Taxi8 | 28/04/2019 | Thursday | 18 | Off-peak | 9.4 | 2.2 | 89 (64 - 113) | 8.77E-05 (1.22E-04 - 6.85E-05) | 1.16E-03 (1.61E-03 - 9.06E-04) |
|  | Taxi9 | 29/01/2019 | Saturday | 26 | Peak | 9 | 1.0 | 190 (129 - 251) | 3.91E-05 (5.76E-05 - 2.96E-05) | 5.17E-04 (7.61E-04 - 3.92E-04) |
| Train | Train1 | 22/06/2019 | Monday | 103 | Peak | 73 | 2.2 | 481 (341 - 621) | 1.26E-04 (1.77E-04 - 9.72E-05) | 1.66E-03 (2.34E-03 - 1.28E-03) |
|  | Train2a | 29/04/2019 | Friday | 71 | Peak | 120 | 1.6 | 1443 (917 - 1968) | 6.88E-05 (1.08E-04 - 5.04E-05) | 9.09E-04 (1.43E-03 - 6.66E-04) |
|  | Train2b | 29/06/2019 | Wednesday | 71 | Peak | 130 | 1.2 | 2469 (2163 - 2775) | 4.35E-05 (4.97E-05 - 3.87E-05) | 5.76E-04 (6.57E-04 - 5.12E-04) |
|  | Train3 | 22/06/2019 | Monday | 64 | Peak | 17 | 1.0 | 232 (192 - 273) | 6.05E-05 (7.31E-05 - 5.15E-05) | 7.99E-04 (9.66E-04 - 6.81E-04) |

## Rebreathed air calculation

We calculated rebreathed value against the lowest CO2 value measured in the 24-hour time period using the following equation for the rebreathed percentage (f) [26]:

$f=\frac{C_{in}-C_{out}}{C_{ex}}\cdot100$

(1)

Where:

f = rebreathed percentage

C_in_ = observed concentration of CO2 in the indoor air

C_out_ = concentration of CO2 in the outdoor air (minimum recorded value from each 24-hour record set)

C_ex_ = concentration of CO2 in the exhaled air (from literature 38,000 ppm, [28])

The rebreathed proportion from other people was then calculated by:

$$f_{o}=\frac{f(n-1)}{n}$$

(2)

Where:

*f_0_* = rebreathed proportion from other people

*f* = rebreathed fraction

n = the number of people recorded at the indoor location

We calculated the Wells-Riley probability of a susceptible person becoming infected using the following equation[6]

$$P=1-e^{-\frac{Iqpt}{Q}}$$

(3)

*P* = probability that a susceptible person becomes infected

*I* = number of infectors (assumed to be 1% of the number of other people present in the space)

*q* = quanta of airborne infection produced per infector per minute

*p* = beathing rate of each susceptible person (m^3^/min)

*Q* = room ventilation rate (m^3^/min)

*t* = time

## Relationship between CO_2_ concentrations and estimated transmission rate


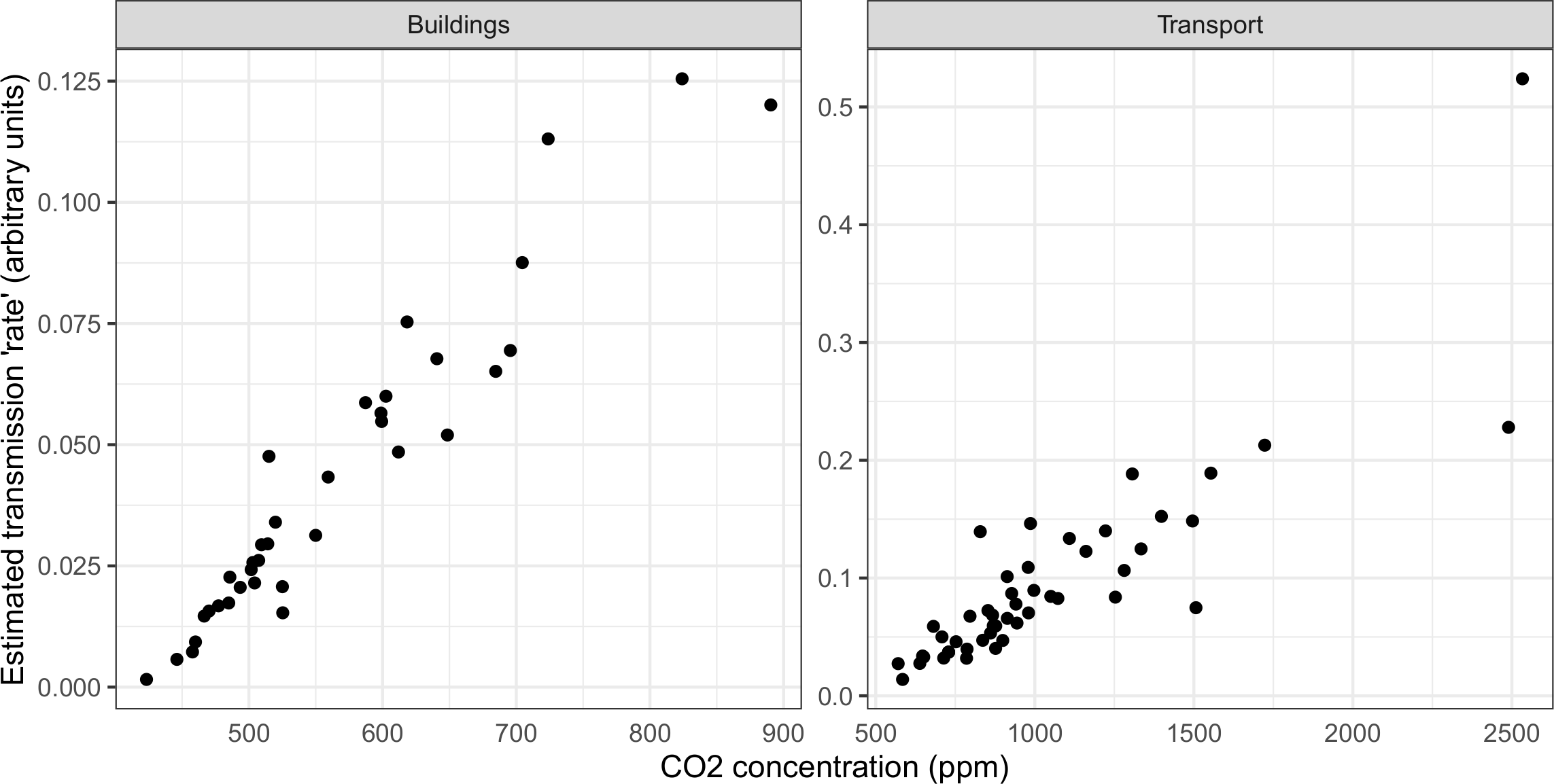


**Figure S1. Estimated transmission rate against mean CO_2_ concentrations by building and vehicle.** Mean CO_2_ concentrations are the mean recorded CO_2_ concentrations during data collection, weighted by the number of people present
